# Somatosensory-Evoked Potentials and Clinical Assessments of Sensory Function Over Time in Patients With Subacute Stroke

**DOI:** 10.1155/np/7939662

**Published:** 2025-01-08

**Authors:** Hiroshi Fuseya, Syoichi Tashiro, Osamu Takahashi, Yukiko Kobayashi, Tetsuya Tsuji, Katsuhiro Mizuno

**Affiliations:** ^1^Department of Rehabilitation Medicine, Shizuoka Cancer Center, Shizuoka, Japan; ^2^Department of Rehabilitation Medicine, School of Medicine, Keio University, Tokyo, Japan; ^3^Department of Rehabilitation Medicine, Faculty of Medicine, Kyorin University, Tokyo, Japan; ^4^Department of Rehabilitation, Ichikawa City Rehabilitation Hospital, Chiba, Japan; ^5^Department of Clinical Neurophysiology, Tokyo Metropolitan Rehabilitation Hospital, Tokyo, Japan; ^6^Department of Rehabilitation Medicine, Tokyo Metropolitan Rehabilitation Hospital, Tokyo, Japan; ^7^Department of Rehabilitation Medicine, School of Medicine, Tokai University, Kanagawa, Japan

## Abstract

**Objective:** To demonstrate the utility of somatosensory evoked potentials (SEPs) following median nerve stimulation for chronological assessment of sensory function in patients with subacute stroke during rehabilitation.

**Design:** Retrospective study.

**Patients:** Forty-seven patients with hemiparesis due to stroke during the subacute phase.

**Methods:** We screened 363 patients who underwent SEP measurements at a rehabilitation hospital. Among them, 47 who underwent SEP measurements within 1 week after admission and at least 2 weeks after the initial assessment were included in this study. Sensorimotor assessments, including the Semmes−Weinstein monofilament test (SWMT), pain sensation, position sensation, two-point discrimination, and Stroke Impairment Assessment Set (SIAS) motor tests simultaneously with SEP measurements were available for 20 of the 47 patients. The relationship between the SEP peak count and each sensorimotor assessment was examined.

**Results:** SEP amplitudes and latencies showed no significant differences between the initial and second assessments (paired *t*-test, *p*  > 0.05). However, the counts of SEP peaks after NI (N20) increased (Wilcoxon signed-rank test, *p*  < 0.05), indicating changes in the SEP waveform. Furthermore, strong correlations were observed between SEP peak counts, stage, and all functional assessments (counts and SWMT, RS = −0.77, *p* < 0.001; counts and pain sensation, RS = −0.71, *p* < 0.001; counts and position sensation, RS = 0.75, *p* < 0.001; counts and two-point discrimination, RS = −0.74, *p* < 0.001; stage and SWMT, RS = −0.74, *p* < 0.001; stage and pain sensation, RS = −0.69, *p* < 0.001; stage and position sensation, RS = 0.74, *p* < 0.001; and stage and two-point discrimination, RS = −0.75, *p* < 0.001; all Spearman's rank correlation coefficients).

**Conclusion:** Despite the limitations of the retrospective study design, our study highlights the utility of SEPs for evaluating sensory function in patients with subacute stroke, setting the foundation for further investigations on the use of SEPs to assess functional changes in patients with subacute stroke undergoing rehabilitation.

## 1. Introduction

Sensory function impairment commonly occurs following stroke [1], and the estimated prevalence of sensory disorders in patients with stroke ranges from 50% to 85% [2]. The extent of recovery from sensory impairment following stroke is related to the severity of the initial deficit and has been reported to require more than 6 months [3, 4]. Assessing sensory function is crucial for evaluating poststroke impairment and predicting functional outcomes [5]. However, this aspect is frequently overlooked during stroke rehabilitation, and parameters such as motor function are often prioritized [5, 6]. Nevertheless, central sensory perception is crucial for motor learning [7]. Several studies have reported that sensory impairment adversely affects rehabilitation outcomes due to the resulting lack of sensory feedback and inhibition of motor learning [7–9]. Moreover, somatosensory impairment has been shown to be significantly associated with upper limb motor recovery and may directly influence activities of daily living [10, 11]. Additionally, sensory deficits predict the efficacy of neurorehabilitation using neuromuscular electrical stimulation in patients with chronic stroke [12]. Although the importance of assessing poststroke sensory deficits in rehabilitation has been recognized, sensory function has not been adequately evaluated in clinical settings [5]. This is because the clinically convenient assessments of sensory dysfunction are less reliable and reproducible than evaluations of motor dysfunction, and it is difficult to accurately assess sensory dysfunction in patients with severe aphasia or other cognitive impairments [13]. Therefore, systematic reviews on stroke rehabilitation have recommended objective assessments of somatosensory function using electrophysiology or neuroimaging [13, 14]. Neurophysiological modalities, such as somatosensory evoked potentials (SEPs), magnetoencephalography, and functional magnetic resonance imaging, can quantitatively and objectively assess sensory deficits [5, 15, 16].

Compared to magnetoencephalography and functional magnetic resonance imaging, SEP evaluation has the advantage of being cost-effective and can be performed for patients with stroke in general clinical settings [17, 18]. SEPs can also assess the functions of the dorsal column of the spinal cord and the medial lemniscus, which are associated with tactile sensation and proprioception [19]. Cortical peaks found after the first negative cortical peak NI (N20) reflect sensorimotor processing [20, 21]. Peaks shorter than 40 ms are believed to be generated in the contralateral area 3b of S1, while those longer than 40 ms are thought to be generated in several areas in addition to area 3b, including areas 1 and 2 [22, 23]. Thus, SEPs are useful for evaluating sensory function by origin [24].

Although some studies have shown a relationship between SEPs and upper limb motor function recovery [14, 25–28], other studies have demonstrated that SEPs alone have limited value in predicting outcomes [29, 30]. Furthermore, studies examining chronological changes in SEPs and sensory function are limited [17, 31, 32]. The loss of SEP waveform is associated with perceptual disturbance [17, 32], and the improvement of abnormal SEPs within 6 months of stroke onset is correlated with clinical improvement [31]. A recent study has suggested that changes in SEP waveforms are associated with improving sensory function through neurorehabilitation and neuromuscular electrical stimulation [33]. Although the relationship between SEPs and cortical plasticity has not been fully elucidated, there appears to be a correlation between assessments of different sensory and motor functions and changes in SEP components [30]. However, most studies have focused on the relationship between SEPs and motor and activity functions; there appears to be no comprehensive study that has specifically evaluated the chronologic relationship between SEP peak counts and sensory function. Therefore, we considered that the correlations between SEP parameters and improvements in multiple sensory assessments remain unclear.

In this study, we aimed to confirm the validity of SEPs in assessing sensory impairment and to demonstrate the potential utility of SEPs in evaluating changes in sensory function following neurorehabilitation. Thus, we chronologically measured SEPs following median nerve stimulation and investigated the changes in SEP waveforms in patients with subacute stroke. Additionally, we compared these findings with the clinical assessments of sensory function.

## 2. Materials and Methods

### 2.1. Ethical Considerations

This study was conducted in accordance with the tenets of the Declaration of Helsinki and was approved by the Ethics Committee of Ichikawa City Rehabilitation Hospital (approval number: 28–7). All patients provided written informed consent before the commencement of the study.

### 2.2. Participants and Study Procedure

In this study, we screened 363 patients admitted to a rehabilitation hospital during the subacute phase of stroke (defined as within 2 months after onset) between January 1, 2000, and December 31, 2002, for whom upper limb SEP data were available. Of these, 47 patients met the following criteria: aged 20–80 years with hemiparesis due to initial supratentorial cerebral infarction or hemorrhage, had their first SEP measured within 1 week after admission (T1), and underwent the second SEP measurement at least 2 weeks after the first test (T2; Group A). Additionally, 20 of the 47 patients had their sensory and motor function evaluated using multiple sensory evaluations: Semmes–Weinstein monofilament test (SWMT) [34], pain sensation using an algometer [35], position sensation through the Stroke Impairment Assessment Set (SIAS) [36, 37], and two-point discrimination [38] as well as SIAS motor tests [36, 37] simultaneously with SEP measurements (Group B). These sensory function assessments were selected for their high reliability and use in clinical and research settings in conjunction with neurophysiological assessments related to various neurological diseases, including stroke [12, 14, 34, 35, 39–41]. The dedicated physiatrist and occupational therapist conducted behavioral assessments (SIAS motor tests, SWMT, pain sensation, position sensation, and two-point discrimination), the results of which were extracted from the patients' medical records. In this study, the behavioral assessments were performed at the therapists' discretion in clinical practice, and SEP data were collected later and reevaluated by two other experts; therefore, blindness in the outcome evaluation was maintained. In this study, we first examined changes that could be evaluated using SEP waveforms alone, utilizing all SEP waveforms of 47 cases (Group A); subsequently, we compared the clinical findings and SEP waveforms using 20 patients (Group B) for whom both data were available. During the hospital stay, all patients underwent daily comprehensive multidisciplinary physical and occupational therapy rehabilitation for 120–180 min, 5 days a week, and were discharged after approximately 3 months.

### 2.3. Electrophysiological Assessment

#### 2.3.1. SEP Recording

Neuropack 8 (Nihon–Kohden, Tokyo, Japan) was used to record the SEPs. According to the methods described in previous studies [17, 18], the active electrodes were positioned over the contralateral frontal cortex (F3 and F4) and bilateral somatosensory cortex (C3′ and C4′, defined as 2 cm behind each of C3 and C4) on the 10−20 International System. SEPs of N13 peaks in the median nerve were assessed using the active electrode at C7 (seventh cervical vertebra). The reference electrodes were placed bilaterally on the ear lobes, and reference values were determined by averaging their signals. The impedance of all electrodes was maintained at <5 kΩ. In the SEP evaluation, the N13 peaks were confirmed in the waveform recorded on C7 in all patients. Since the N13 peak originates from the cervical spinal cord [42], we confirmed that the patient had no remarkable spinal cord lesion.

While recording the SEPs, the median nerves were stimulated superficially at the wrist using a square wave with a duration of 0.2 ms. The stimulation intensity was set to induce a visible yet minimal muscular contraction of the abductor pollicis brevis muscle (~6–10 mA). The wrist was alternately stimulated at a frequency of 2.05 Hz (i.e., 1.025 Hz on each side). Signals were recorded from −20 to 100 ms for each pulse, digitized at a sampling frequency of 5000 Hz, and filtered through a band-pass of 2–2000 Hz. To ensure reproducibility, data for analysis were calculated as an average of two independent trials obtained for 1000 stimuli using the addition-averaging method [17, 18, 21].

#### 2.3.2. Analysis of SEP Waveforms

First, the N13 peak latency was assessed at the C7 spinal level (reference: earlobe). Second, to count the number of cortical SEP peaks and assess the somatosensory area, the P0 (P14), NI (N20), PI (P24), NII (N33), PII (P45), and NIII (N60) peak parameters were evaluated at C3′ and C4′. Two clinical neurophysiology experts (a clinical technologist and a board-certified physiatrist) blinded to the patients' clinical information manually judged and measured the presence of SEP peaks and their parameters. Two experts, with reference to the SEP peaks on each nonparetic side, independently determined the presence of SEP peaks. Any disagreements between the experts were resolved through discussion. The average value of these two measurements was used in the analyses. Furthermore, the counts of SEP peaks were recorded based on the presence of the following five peaks: NI, PI, NII, PII, and NIII, and the SEP waveform was classified into three stages based on the counts of peaks. We classified peak counts of 0, 1–4, and 5 as stages I (absence), II (incomplete), and III (complete), respectively.

In amplitude, the P0-NI amplitudes were recorded as a reference, and the value of 0 was applied when the NI peak could not be determined. Subsequently, the interpeak latencies between each peak and the NI latencies were calculated. The N13-NI latency was considered the central conduction time (CCT), representing the pure conduction time within the somatosensory pathways of the brainstem and cerebrum [43, 44].

### 2.4. Behavioral Assessments

#### 2.4.1. SWMT

The SWMT (Sakai Medical Co., Ltd., Tokyo, Japan) was used to assess the tactile sensory threshold. In this test, monofilaments were perpendicularly pushed against the palmar surface of the index finger with the patients in a supine position and their eyes closed [45]. Force was sufficiently applied until the filament was bent or twisted, and the patients were then asked if they could sense the monofilament on their index finger. If the patients could not feel the monofilament more than once, the filament strength was judged to be below the threshold, and the size number of the monofilament was then recorded. If the patient could not sense any of the filaments, the size number was assumed to be “7.” Target forces ranging from 0.008 to 300 g can be measured using this method, depending on the monofilament size. Furthermore, the natural logarithm of the target force is transformed into the monofilament number [46]. The SWMT was performed three times by the same examiner for each patient [34].

#### 2.4.2. Pain Sensation

An algesimeter (Yufu Seiki Co., Ltd., Tokyo, Japan), capable of weighing pressures ranging from 1 to 20 g, was used to measure the pain threshold following a previously mentioned protocol [35]. The index finger was stimulated with an algesimeter, and the value at which pain was felt was recorded. When the patient did not feel pain at the maximum pressure, the value was assumed to be 21 g.

#### 2.4.3. Position Sensation

Proprioception of the upper extremity was assessed using the SIAS [36, 37] and scored between 0 (sensory deficit) and 3 (no remarkable impairment). Position disturbances were evaluated based on the accuracy of passive joint movements at the index finger or thumb.

#### 2.4.4. Two-Point Discrimination

Two-point discrimination was examined according to the protocol of Mackinnon and Dellon [38] by lightly touching the palmar surface of the patient's hand with a caliper (Disk-Criminator; Sakai Medical Co., Ltd.). Testing was initiated with a 5-mm distance between the two points of the caliper, and patients were asked whether they could feel one or two different points. If they could recognize two points, the distance of the caliper gradually decreased until the patient could no longer feel two distinct points, which was defined as the two-point discrimination threshold. If the patient could not feel two distinct points in the first step, the distance of the caliper gradually increased until the patient could feel two distinct points. They had distances of 2, 3, 4, 5, 6, 7, 8, 9, 10, 11, 12, 13, 14, 15, and 20 mm.

#### 2.4.5. Motor Function

Motor function was assessed using the SIAS motor tests [36, 37]. Upper extremity function was assessed using the knee-mouth and finger function tests, which were scored between 0 (complete paralysis) and 5 (no remarkable impairment). The knee-mouth test was used to assess the ability of the proximal upper extremity to raise the paretic arm from the knee to the mouth. The finger function test was used to evaluate the ability of the distal upper extremity to flex the fingers separately from the thumb to the little finger and extend separately from the little finger to the thumb. Since score 1 of the SIAS finger function test is classified into 1a, 1b, and 1c, scores 0–5 were converted to 0–7 sequentially for analysis in the current study.

### 2.5. Statistical Analyses

All data were presented as the mean (standard error of the mean). Paired *t*-tests were used to compare the latencies of each SEP peak and interpeak latencies after NI and P0-NI amplitudes at T1 and T2 on the paretic and nonparetic sides. Wilcoxon signed-rank tests were used to compare the impairments and counts of SEP peaks between T1 and T2. SEP peak count and each sensorimotor assessment was checked for the presence of normally distributed data using the Shapiro–Wilk test. The correlation between the SEP peak count and assessments was examined using the Pearson correlation coefficient in cases where the distribution was normal, and Spearman's rank correlation coefficient in cases where the distribution was not normal. In this analysis, data from T1 and T2 for the same patient were analyzed as separate data sets; the 20 data sets for T1 and 20 data sets for T2 were combined, resulting in a total of 40 data sets. Statistical significance was set at *p*  < 0.05 for all analyses. All analyses were performed using JMP 15 (SAS Institute Inc., Cary, NC, USA).

## 3. Results

### 3.1. Patient Characteristics and Functional Changes

The characteristics of all patients (*N* = 47, Group A) and the fraction of those who underwent sensory assessments (*N* = 20, Group B) are summarized in Table 1. In 20 patients, functional changes were compared bilaterally using clinical assessment scores between T1 and T2. On the paretic side, significant improvements were observed in the SIAS motor function and sensory assessments, excluding position sensation (Table 2; *N* = 20, Group B, Wilcoxon signed-rank tests). Improvements were also found on the nonparetic side; however, this was only in the context of two-point discrimination (Table S1). Sensory evaluation on the nonparetic side was examined to confirm the reproducibility of the SEP measurements. SEPs were not measured in patients with severe aphasia and disorders of consciousness, although they were not excluded during the screening process. This may be because stable SEP results cannot be obtained if the patient does not fully understand the measurement procedures and/or due to other clinical reasons.

### 3.2. Chronological Changes in the Parameters of SEPs

**Figure 1:**
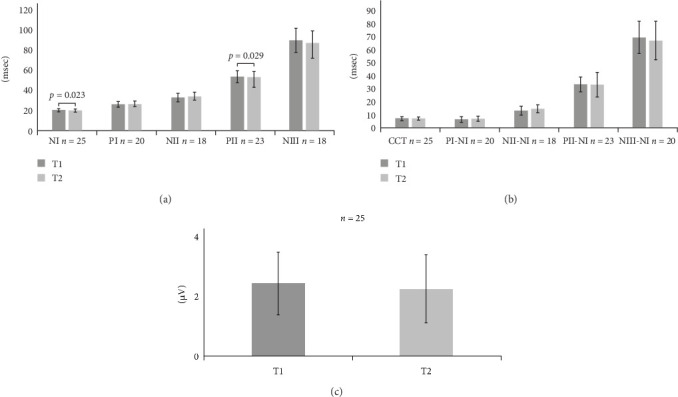
Latencies and amplitude of cortical peaks after NI at T1 and T2 on the paretic side (Group A). (a) Graph showing latencies of cortical peaks after NI at T1 and T2 on the paretic side. Values of peaks observed only at T2 are not included. Significant differences are detected in NI and PII *⁣*^*∗*^*t*-test). (b) Graph showing interpeak latencies at T1 and T2 on the paretic side. No significant difference is detected (*t*-test). (c) Graph showing P0-NI amplitude at T1 and T2 on the paretic side. No significant difference is detected (T1:2.45 (1.07), T2:2.26 (1.15), *p*=0.68; *t*-test). All data are presented as mean (standard error of the mean). CCT, central conduction time; NI, N20; NII, N33; NIII, N60; PI, P24; PII, P45; T1, initial assessment; T2, second assessment.

Significant differences were detected in the NI and PII peak latencies after NI between T1 and T2 on the paretic side (NI, T1:20.24 (1.48), T2:19.91 (1.47), *p*=0.023; PII, T1:53.7 (5.83), T2:53.12 (9.87), *p*=0.029; (*t*-test); Figure 1a and Table S2, Group A). Neither interpeak latencies nor the P0-NI amplitude differed between T1 and T2 on the paretic side (Figure 1b,c and Table S3, Group A). On the nonparetic side, a significant difference was found in NI peak latency (T1:19.23 (1.15), T2:19.04 (1.09), *p*=0.021); however, no significant differences were detected in other peak latencies, peak-to-peak latencies, or P0-NI amplitude between T1 and T2 (Figure S1a–c, Tables S4 and S5, Group A).

**Figure 2:**
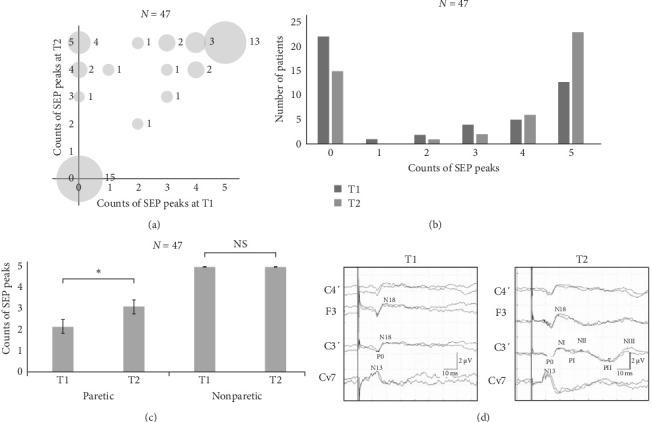
Transition of SEP peak counts from T1 to T2 in 47 patients (Group A). (a) Scatter plot showing the correlation between peak counts at T1 and T2 on the paretic side. (b) Graph showing the transition of a number of patients in each count of peaks from T1 to T2 on the paretic side. (c) Graph showing the average counts of median nerve SEP peaks after NI at T1 and T2. A significant increase is observed in the counts of SEP peaks (*⁣*^*∗*^: T1, 2.17 (0.33); T2, 3.13 (0.33); *p*  < 0.001; Wilcoxon signed-rank tests). All SEP peaks are preserved on the nonparetic side. All data are presented as mean (standard error of the mean). (d) Representative median nerve SEP waves in the same patient throughout the study period. Although no cortical peak is detected at T1, all cortical peaks are restored at T2. NI, N20; SEP, somatosensory evoked potential; T1, initial assessment; T2, second assessment.

While the appearance of SEP peaks at T1 was insufficient, it remarkably restored at T2 with a significant change of the cortical peak count in the comparison of pre- and postinpatient rehabilitation (Figure 2a–2c and Table S6, Group A). Representative SEP waves of the median nerve are shown in Figure 2d, demonstrating that the restoration of NI, PI, NII, PII, and NIII peaks occurred at T2 rather than T1. The data classified into SEP stages showed a similar result to that of the SEP peak counts, indicating that SEP staging could be alternatively used to assess recovery in SEPs (Figure S2a,b and Table S7, Group A). Finally, all SEP peaks were preserved on the nonparetic side. The peak count was restored (*N* = 15) or maintained (*N* = 32), including those with full peaks at T1. Thus, none of the patients experienced a peak loss. The statistical significance was maintained when only a subset of patients who underwent sensory testing, specifically Group B, were analyzed (*N* = 20; Figure S3).  Table 3 (Group B) summarizes the presence of SEP peaks in all patients at both time points.

### 3.3. Relationship Between SEP Waveforms and Sensory Functional Recovery

**Figure 3:**
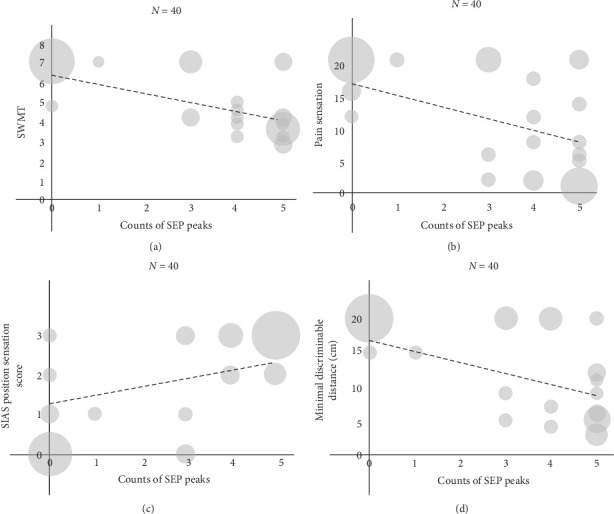
Scatter plots showing the correlation between SEP peak counts and each sensory assessment (Group B). Strong correlations are observed between SEP peaks and (a) SWMT (*ρ* = −0.77, *p*  < 0.001; Spearman's rank correlation coefficient), (b) pain sensation (*ρ* = −0.71, *p*  < 0.001; Spearman's rank correlation coefficient), (c) SIAS position sensation (*ρ* = 0.75, *p*  < 0.001; Spearman's rank correlation coefficient), and (d) two-point discrimination (*ρ* = −0.74, *p*  < 0.001; Spearman's rank correlation coefficient). SEP, somatosensory evoked potential; SIAS, Stroke Impairment Assessment Set; SWMT, Semmes–Weinstein monofilament test.

We analyzed the relationship between SEP waveforms and functions in the patients who underwent behavioral assessments (Group B) at T1 and T2. Since none of the variables were normally distributed (*p*  < 0.01, Shapiro–Wilk test), the correlation analyses were calculated using Spearman's rank correlation coefficient. The correlation between the SEP peak count and each functional sensorimotor assessment (SWMT, pain sensation, SIAS position sensation, two-point discrimination, and SIAS motor scores) was evaluated at T1 and T2 (*N* = 40; we investigated whether a correlation existed between SEP peak counts and sensory functions when evaluated simultaneously, and we assessed T1 and T2 of 20 patients as separate data, resulting in 40 data sets; Group B), which were represented via scatter plots (Figure 3 and Figure S4, Group B). Strong correlations were observed for all sensory assessments except the SIAS knee-mouth test, for which the correlation was moderate (SWMT, RS = −0.77, *p*  < 0.001; pain sensation, RS = −0.71, *p*  < 0.001; position sensation, RS = 0.75, *p*  < 0.001; two-point discrimination, RS = −0.74, *p*  < 0.001; SIAS knee-mouth test, RS = 0.44, *p*=0.004; and SIAS finger test, RS = 0.22, *p*=0.178, Spearman's rank correlation coefficient; Table 4, Group B). The relationship between the functional assessments and SEP stages at T1 and T2 matched the correlation strengths identified with SEP peaks (SWMT, RS = −0.74, *p*  < 0.001; pain sensation, RS = −0.69, *p*  < 0.001; position sensation, RS = 0.74, *p*  < 0.001; two-point discrimination, RS = −0.75, *p*  < 0.001; SIAS knee-mouth test, RS = 0.41, *p*=0.008; and SIAS finger test, RS = 0.18, *p*=0.25, Spearman's rank correlation coefficient; Table S8 and Figure S5, Group B).

## 4. Discussion

In this study, we first comprehensively examined the temporal changes in SEP peak latencies, amplitudes, and waveforms in 47 patients with stroke who underwent intensive rehabilitation during the subacute phase (Group A). SEP peak latencies were significantly shortened between T1 and T2 in NI and PII on the paretic side and in NI on the nonparetic side. These changes are believed to reflect changes in the condition of patients with subacute strokes, such as a reduction in cerebral edema around lesions. However, the differences were <0.4 ms on average, which are relatively small compared to those of the standard deviation of the nonparetic side (~1.1 ms at both T1 and T2), and thus were deemed clinically insignificant. As in previous studies [47], no clinically important differences were found in SEP peak latencies, and no significant difference was detected in P0-NI amplitude or interpeak latencies, including CCT between T1 and T2. These findings suggested that no remarkable changes were induced in simple SEP parameters after conventional rehabilitation during the subacute phase of stroke.

In contrast, the SEP peak count was significantly restored over the same period. Additionally, the relationship between SEP peak counts and neurological findings of sensory function (SWMT, pain sensation, position sensation, and two-point discrimination) was investigated in 20 patients (Group B), and significant correlations were detected between SEP peak counts and sensory function. The transition of the number of patients in each SEP peak count and stage on the paretic side from T1 to T2 indicated little improvement in patients with SEP counts 1−4 and stage II. Therefore, peak counts 1−4 were grouped into stage II since the evaluation based on the SEP stage revealed similar trends.

These results indicate that SEP waveform analysis could be a useful method to evaluate the chronology of sensory cortical recovery in patients with stroke. Given that the parameters of hidden peaks could not be measured, such parameters are not always appropriate to compare the total features of previous and subsequently recorded SEPs. As SEPs recover over time, hidden peaks appear, and their parameters become incorporated in the mean values [31]. On the other hand, the type of SEP waveform conveys information about the presence or absence of peaks and can be determined for all individuals. Therefore, analysis of SEP waveform types will sometimes be more practical and facilitate an intuitive understanding of SEPs.

Although some studies have investigated SEP changes in subacute stroke [27–29], this study uniquely compared clinical sensory assessments and SEP waveforms in patients with subacute stroke, clarifying their relationship. We observed significant improvements in sensory function throughout the observation period. These improvements were accompanied by a rise in SEP peak counts, indicating that SEPs and sensory functions were positively correlated. It is recognized that sensory impairment tends to improve over time during the acute and subacute phases of stroke [4].

SEPs reflect structural damage to the sensorimotor pathway [48, 49]. It has been reported that abnormal SEP waveforms reflect dysfunction of the medial lemniscus pathway at the origin of invalid peaks [50]. Sensory functions associated with the medial lemniscus pathway include tactile, positional, and two-point discrimination sensations [51]. Our study revealed strong correlations between SEP peak counts and the outcomes of sensory assessments (SWMT, pain sensation, position sensation, and two-point discrimination) in the context of these parameters. Additionally, SEP peaks were correlated with pain sensations despite being transmitted over extra-lemniscal pathways. A possible explanation for this may be that pain signals are processed in the primary and secondary somatosensory cortices [51, 52], and pain pathways partly converge with other somatosensory pathways at the thalamocortical level [19], which are included in the pathway evaluated by SEPs. Furthermore, laser-evoked potentials are event-related potentials specialized for pain evaluation; laser pulses specifically activate the nociceptive and thermoreceptive pathways comprising the anterolateral spinothalamic tract in the spinal cord and brainstem [19, 52]. However, in thalamocortical lesions such as those evaluated in the current study, the somatosensory pathway largely converges in the common thalamic nucleus; consequently, the distinction between laser-evoked potentials and SEPs is not entirely clear [19]. Thus, it is unsurprising that this study showed correlations between SEP peaks and pain sensations.

Moreover, in this study, we found no change in CCT between T1 and T2. This means that no relationship was found between CCT changes and sensory function recovery during the stroke rehabilitation period. CCT represents impulse propagation from the dorsal column nuclei to the cortex [44] and is prolonged in comatose patients or those with cerebrovascular diseases [43, 53]. In the context of our findings, the evaluation of CCT could have been hindered by the lack of the NI peak in 22 of the 47 patients at T1, resulting in a lack of correlation between CCT and recovery. Additionally, the evaluation of latencies, including peak and interpeak latencies, showed no clinically remarkable changes in our study. SEP peak latencies, including CCT, are reported to be influenced by peripheral nerve conduction time [44], which may complicate their use as a criterion for sensory assessment.

Although no change was observed in the P0-NI amplitude in our study, previous studies have reported the effect of rehabilitative interventions on increasing SEP amplitude. For example, it has been previously demonstrated that high-frequency somatosensory stimuli may induce an increased NI-PI amplitude and improve behavioral perception in healthy controls [20]. A home exercise program, combined with botulinum toxin type A injections, has been reported to reduce spasticity, increase the functional use of paretic limbs, and increase the NI amplitude [54]. Furthermore, a study showed that increased cortical peak counts and improved SEP peak amplitudes can be elicited by neurorehabilitation in patients with chronic stroke [33]. In this study, we measured the P0-NI amplitude as one of the reference measurements, and no changes were observed. The absence of SEP amplitude changes can be explained by the considerable variability in P0-NI amplitude, which is sometimes greater on the paretic side than on the nonparetic side. This study's findings suggest that the SEP amplitude is not easily evaluated as an indicator of neurological changes during the early phases of stroke.

SEPs may enable an objective assessment of medial lemniscus sensory disorders in patients with stroke. While a detailed assessment of sensory impairment has been shown to help predict motor functional outcomes, activities of daily living, and activity participation in stroke survivors [6, 10, 55, 56], we believe that SEPs could become another information source of sensory functionality to guide the clinical management of stroke. As shown in our current study, the SEP cortical peak count could represent an easy and reliable method to interpret SEPs as opposed to the latency and amplitude of those peaks. Thus, evaluating SEP waveforms can provide an objective and intuitive assessment of sensory impairment, which may be useful in predicting rehabilitation outcomes.

This study had some limitations. First, it was a retrospective observational study limited to a single rehabilitation institute that included only 47 patients (Group A) with some missing data, as shown in Table 1, and only 20 with data to show a correlation between sensory deficits and SEP peaks (Group B). Although this number of patients may be somewhat small, we considered it satisfactory to assess changes in SEP waveforms in patients with subacute stroke (*n* = 47) and to estimate the correlations between these changes and sensory deficits (*N* = 20, assessed T1 and T2 of 20 patients as separate data, resulting in 40 data sets). A power analysis of Spearman's rank correlation coefficient was calculated with *N* = 40, a significance level of 5%, and an expected correlation coefficient of 0.7, resulting in 0.997 (SPSS ver. 28.010). Therefore, although the sample size was small, it appears possible to predict the relationship between SEPs and sensory impairment. However, the results of the current study cannot be directly extrapolated to all patients with stroke. To confirm the findings of the current study, prospective studies with increased sample sizes and additional measurement points are needed. Similarly, the small number of patients may have been one of the reasons why no difference was identified between T1 and T2 in the SEP parameters. However, in patients with subacute stroke, parameters such as amplitudes and latencies are missing or vary widely between patients; therefore, even in a study that includes a large number of patients it may be challenging to obtain statistically significant differences [47]. Second, the results of the current study may not be generalizable to all patients with stroke, as those with severe aphasia and disorders of consciousness were excluded from the screening process. SEPs were not measured within 1 week after admission in patients with severe aphasia and disorders of consciousness because there was a possibility that stable SEP results could not be obtained if the patient did not fully understand the measurement procedures. Although SEP waveform patterns may enable an objective assessment of sensory function in patients with stroke, further studies are needed to determine whether such an approach can be applied to patients who are unable to cooperate, such as those with severe aphasia or disorders of consciousness. Third, in this study, the male/female and left/right hemiparetic ratios differed between all 47 patients and the 20 who underwent sensory assessments. These differences may have influenced the results. Fourth, a detailed peripheral nerve evaluation was not performed. Since the N13 peak could be measured, the stimulus was deemed to have reached the brain stem, indicating no severe peripheral neuropathy. Finally, tibial nerve SEPs were not evaluated in the current study. Although some patients had undergone SEP measurements involving their tibial nerves, they were excluded from this study because there were a few patients for whom data for both upper and lower extremities were available; this was due to the difficulty in maintaining appropriate posture for tibial nerve SEPs, particularly in cases with severe neurological impairment. It has been reported that balance function can be evaluated using tibial nerve SEPs [57], and it may also be possible to assess the sensory deficits of the lower limbs, similar to how sensory deficits of the upper limbs were evaluated in the current study. Further research using lower limb SEPs is required to reinforce the utility of SEPs for patients with subacute stroke.

## 5. Conclusions

The findings of this study suggest that the analysis of SEP waveforms is a feasible and objective sensory assessment tool to evaluate chronologic changes in our sample of patients with subacute stroke. The SEP peak count strongly correlates with sensory function, indicating the need for further investigations to assess the functional changes of subacute stroke rehabilitation via SEPs. Generalizability of our results to all patients with subacute stroke would require prospective studies using SEP of the upper and lower limbs, combining a variety of sensory tests, increasing the sample size, adding measurement points, and examining the brain site of lesion, as well as including patients with severe aphasia or disorders of consciousness. Our study highlighted the utility of measuring SEPs in patients with subacute stroke to evaluate sensory function, which is crucial for stroke rehabilitation.

## Figures and Tables

**Table 1:** Patient characteristics.

Parameters	Results	
	*N* = 47, Group A	*N* = 20, Group B
Age (years)	60.6 (9.0)	57.7 (8.1)
Sex	Female (*N* = 14), male (*N* = 33)	Female (*N* = 5), male (*N* = 15)
Hemiparetic side	Left (*N* = 25), right (*N* = 22)	Left (*N* = 15), right (*N* = 5)
Diagnosis	Data not available	Ischemia (*N* = 8), hemorrhage (*N* = 12)
Site of lesions	Data not available	Putamen (*N* = 5), thalamus (*N* = 7), internal capsule (*N* = 1), corona radiata (*N* = 2), middle cerebral artery (*N* = 4), posterior cerebral artery (*N* = 1)
No. of days from onset to T1	Data not available	30.4 (10.0)
No. of days from T1 to T2	44.5 (25.5)	41.9 (8.2)

*Note:* All data are presented as mean (standard error of the mean). T1, initial assessment; T2, second assessment.

**Table 2:** Functional changes observed on the paretic side (Group B).

Sensorimotor assessments	T1	T2	*p*-Value
SWMT number	5.71 (0.33)	4.95 (0.39)	0.003
Pain sensation	13.8 (1.98)	10.95 (1.90)	0.042
Position sensation (SIAS)	1.65 (0.92)	1.85 (0.30)	0.163
Two-point discrimination	15.15 (1.37)	12.05 (1.71)	0.021
SIAS knee-mouth	1.85 (0.29)	2.55 (0.26)	0.001
SIAS finger	1.30 (0.33)	1.90 (0.35)	0.015

*Note:* All data are presented as mean (standard error of the mean). T1, initial assessment; T2, second assessment.

Abbreviations: SIAS, Stroke Impairment Assessment Set; SWMT, Semmes–Weinstein monofilament test.

**Table 3:** Status of somatosensory evoked potential (SEP) peaks (Group B).

Case	T1					T2				
	NI	PI	NII	PII	NIII	NI	PI	NII	PII	NIII
1	−	−	−	−	−	−	−	−	−	−
2	−	−	−	−	−	−	−	−	−	−
3	−	−	−	−	−	−	−	−	−	−
4	−	−	−	−	−	−	−	−	−	−
5	−	−	−	−	−	−	−	−	−	−
6	−	−	−	−	−	+	−	−	+	+
7	−	−	−	−	−	+	+	+	+	−
8	−	−	−	−	−	+	+	+	+	+
9	−	−	−	−	−	+	+	+	+	+
10	+	−	−	−	−	+	+	+	+	−
11	+	−	−	+	+	+	−	−	+	+
12	+	−	−	+	+	+	+	+	+	+
13	+	−	−	+	+	+	+	+	+	+
14	+	+	+	+	-	+	+	+	+	+
15	+	+	+	+	-	+	+	+	+	−
16	+	+	+	+	+	+	+	+	+	+
17	+	+	+	+	+	+	+	+	+	+
18	+	+	+	+	+	+	+	+	+	+
19	+	+	+	+	+	+	+	+	+	+
20	+	+	+	+	+	+	+	+	+	+

*Note:* NI, N20; NII, N33; NIII, N60; PI, P24; PII, P45; T1, initial assessment; T2, second assessment.

**Table 4:** Correlation between SEP peak counts and sensorimotor assessments (Group B).

Sensorimotor assessments	Correlation coefficient	*p*-Value
SWMT number	RS = −0.77	<0.001
Pain sensation	RS = −0.71	<0.001
Position sensation (SIAS)	RS = 0.75	<0.001
Two-point discrimination	RS = −0.74	<0.001
SIAS knee-mouth test	RS = 0.44	0.004
SIAS finger test	RS = 0.22	0.178

Abbreviations: SEP, somatosensory evoked potential; SIAS, Stroke Impairment Assessment Set; SWMT, Semmes–Weinstein monofilament test.

## Data Availability

The spreadsheet data used to support the findings of this study are available from the corresponding author upon request.

## Supporting Information

Supporting Information Figure S1. Latencies and amplitude of cortical peaks after NI at T1 and T2 on the nonparetic side (Group A). (a) Graph showing latencies of cortical peaks after NI at T1 and T2 on the nonparetic side. A significant difference is detected in NI (*⁣*^*∗*^*t*-test). (b) Graph showing interpeak latencies at T1 and T2 on the nonparetic side. No significant difference is detected (*t*-test). (c) Graph showing P0-NI amplitude at T1 and T2 on the nonparetic side. No significant difference is detected (T1: 3.96 (1.79), T2: 3.73 (1.81), *p*=0.064; *t*-test). Figure S2. Transition of somatosensory evoked potential (SEP) stage from T1 to T2 in 47 patients (Group A). (a) Scatter plot showing the correlation between SEP stage at T1 and T2 on the paretic side. (b) Graph showing the transition of a number of patients in each SEP stage from T1 to T2 on the paretic side. Figure S3. Features of SEPs in patients who underwent clinical assessment (Group B). Figure S4. Scatter plot showing the correlation between SEP peak counts and each SIAS motor assessment (Group B). (a) A moderate correlation is observed between SEP peaks and proximal upper limb motor function (*ρ* = 0.44, *p*=0.004; Spearman's rank correlation coefficient). (b) No correlation is observed between SEP peaks and distal upper limb motor function (*ρ* = 0.22, *p*=0.178; Spearman's rank correlation coefficient). Figure S5. Scatter plots showing the correlation between the SEP stage and sensorimotor assessment (Group B). Strong correlations are observed between (a) SEP stage and SWMT (*ρ* = −0.74, *p* < 0.001; Spearman's rank correlation coefficient), (b) pain sensation (*ρ* = −0.69, *p* < 0.001; Spearman's rank correlation coefficient), (c) SIAS position sensation (*ρ* = 0.74, *p* < 0.001; Spearman's rank correlation coefficient), and (d) two-point discrimination (*ρ* = −0.75, *p* < 0.001; Spearman's rank correlation coefficient). (e) A moderate correlation is found between the SEP stage and proximal upper limb motor function (*ρ* = 0.41, *p*=0.008; Spearman's rank correlation coefficient). (f) No correlation is observed between the SEP stage and distal upper limb motor function (*ρ* = 0.18, *p*=0.25; Spearman's rank correlation coefficient). Table S1. Functional changes observed on the nonparetic side (Group B). Table S2. Latencies of cortical peaks at T1 and T2 on the paretic side. Table S3. Interpeak latencies at T1 and T2 on the paretic side. Table S4. Latencies of cortical peaks at T1 and T2 on the nonparetic side. Table S5. Interpeak latencies at T1 and T2 on the nonparetic side. Table S6. Transition of a number of patients in each count of peaks from T1 to T2 on the paretic side. Table S7. Transition of a number of patients in each SEP stage from T1 to T2 on the paretic side. Table S8. Correlation between SEP stage and sensorimotor assessments (Group B).
