# Correction to “Somatosensory‐Evoked Potentials and Clinical Assessments of Sensory Function Over Time in Patients With Subacute Stroke”

**DOI:** 10.1155/np/9805451

**Published:** 2026-01-18

H. Fuseya, S. Tashiro, O. Takahashi, Y. Kobayashi, T. Tsuji, and K. Mizuno, “Somatosensory‐Evoked Potentials and Clinical Assessments of Sensory Function Over Time in Patients With Subacute Stroke,” *Neural Plasticity* 2025, no. 1 (2025): 1–12, https://doi.org/10.1155/np/7939662.

In the article titled “Somatosensory‐Evoked Potentials and Clinical Assessments of Sensory Function Over Time in Patients With Subacute Stroke”, the authors wish to provide clarifications to the article’s ethical considerations in Section 2.1.

Section 2.1. Ethical Considerations should read:

“This study was conducted in accordance with the tenets of the Declaration of Helsinki and was reviewed and approved by the institutional ethics committee of Ichikawa City Rehabilitation Hospital (Approval Number: 28–7), the facility where the study was conducted. Because this study was a retrospective analysis based on existing patient medical record data, we used the opt‐out approach and the requirement for written informed consent was waived. All patient data were fully anonymized prior to analysis.”

We apologize for this error.

