# Supplementary material for: Somatosensory-Evoked Potentials and Clinical Assessments of Sensory Function Over Time in Patients With Subacute Stroke
Source: Neural Plast. 2025 Jan 8;2025:7939662. doi: 10.1155/np/7939662 (PMC11735060; doi:10.1155/np/7939662)
Supplement: Supporting Information — Figure S1. Latencies and amplitude of cortical peaks after NI at T1 and T2 on the nonparetic side (Group A). (a) Graph showing latencies of cortical peaks after NI at T1 and T2 on the nonparetic side. A significant difference is detected in NI (⁣∗t-test). (b) Graph showing interpeak latencies at T1 and T2 on the nonparetic side. No significant difference is detected (t-test). (c) Graph showing P0-NI amplitude at T1 and T2 on the nonparetic side. No significant difference is detected (T1: 3.96 (1.79), T2: 3.73 (1.81), p=0.064; t-test). Figure S2. Transition of somatosensory evoked potential (SEP) stage from T1 to T2 in 47 patients (Group A). (a) Scatter plot showing the correlation between SEP stage at T1 and T2 on the paretic side. (b) Graph showing the transition of a number of patients in each SEP stage from T1 to T2 on the paretic side. Figure S3. Features of SEPs in patients who underwent clinical assessment (Group B). Figure S4. Scatter plot showing the correlation between SEP peak counts and each SIAS motor assessment (Group B). (a) A moderate correlation is observed between SEP peaks and proximal upper limb motor function (ρ = 0.44, p=0.004; Spearman's rank correlation coefficient). (b) No correlation is observed between SEP peaks and distal upper limb motor function (ρ = 0.22, p=0.178; Spearman's rank correlation coefficient). Figure S5. Scatter plots showing the correlation between the SEP stage and sensorimotor assessment (Group B). Strong correlations are observed between (a) SEP stage and SWMT (ρ = −0.74, p < 0.001; Spearman's rank correlation coefficient), (b) pain sensation (ρ = −0.69, p < 0.001; Spearman's rank correlation coefficient), (c) SIAS position sensation (ρ = 0.74, p < 0.001; Spearman's rank correlation coefficient), and (d) two-point discrimination (ρ = −0.75, p < 0.001; Spearman's rank correlation coefficient). (e) A moderate correlation is found between the SEP stage and proximal upper limb motor function (ρ = 0.41, p=0.008; Spearman's ra [file 7939662.f1.docx]

**Supplementary Figure Legends**


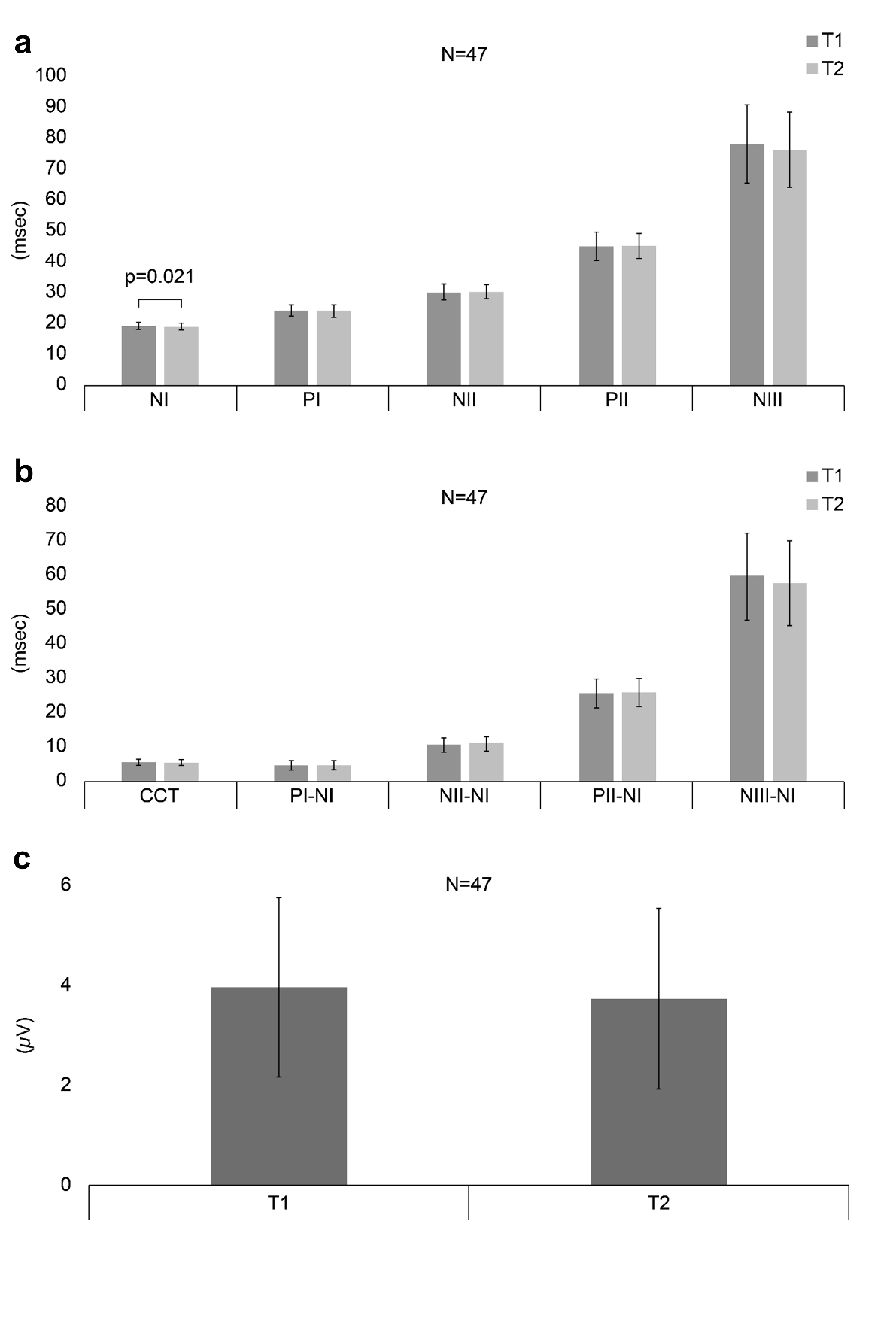


Figure S1: Latencies and amplitude of cortical peaks after NI at T1 and T2 on the nonparetic side (Group A)

(a) Graph showing latencies of cortical peaks after NI at T1 and T2 on the nonparetic side. A significant difference is detected in NI [* *t*-test].

(b) Graph showing interpeak latencies at T1 and T2 on the nonparetic side. No significant difference is detected [*t*-test].

(c) Graph showing P0-NI amplitude at T1 and T2 on the nonparetic side. No significant difference is detected (T1: 3.96 (1.79), T2: 3.73 (1.81), P=0.064; *t*-test).

All data are presented as mean ± standard error of the mean.

NI: N20, PI: P24, NII: N33, PII: P45, NIII; N60, CCT: Central conduction time; T1: initial assessment, T2: second assessment


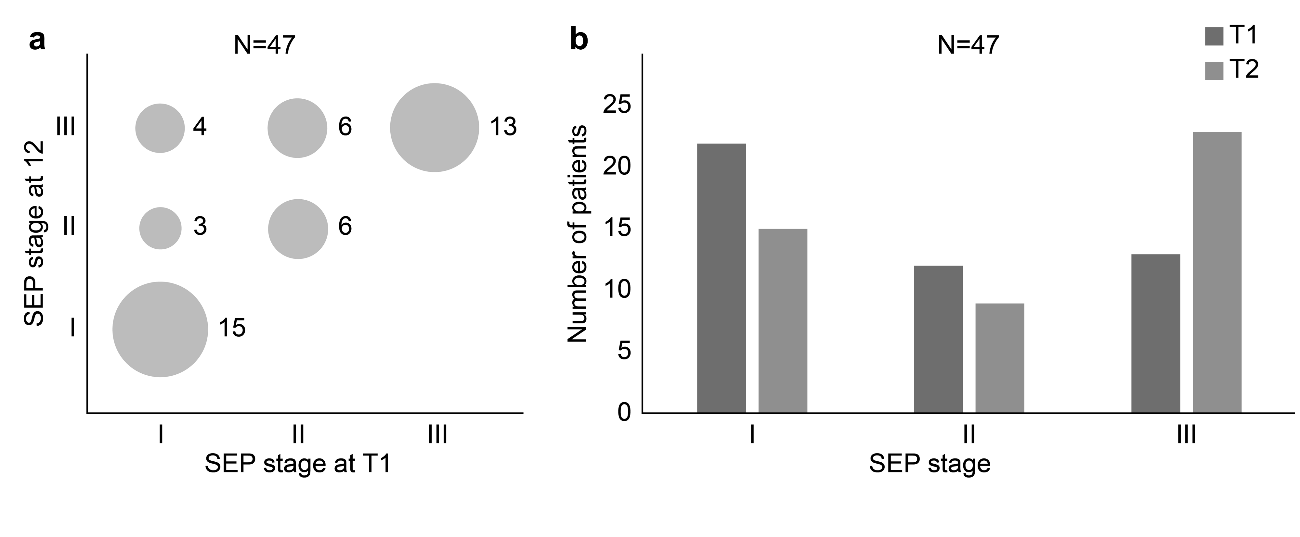


Figure S2: Transition of SEP stage from T1 to T2 in 47 patients (Group A)

(a) Scatter plot showing the correlation between SEP stage at T1 and T2 on the paretic side

(b) Graph showing the transition of a number of patients in each SEP stage from T1 to T2 on the paretic side.

SEP: somatosensory evoked potential, T1: initial assessment, T2: second assessment.


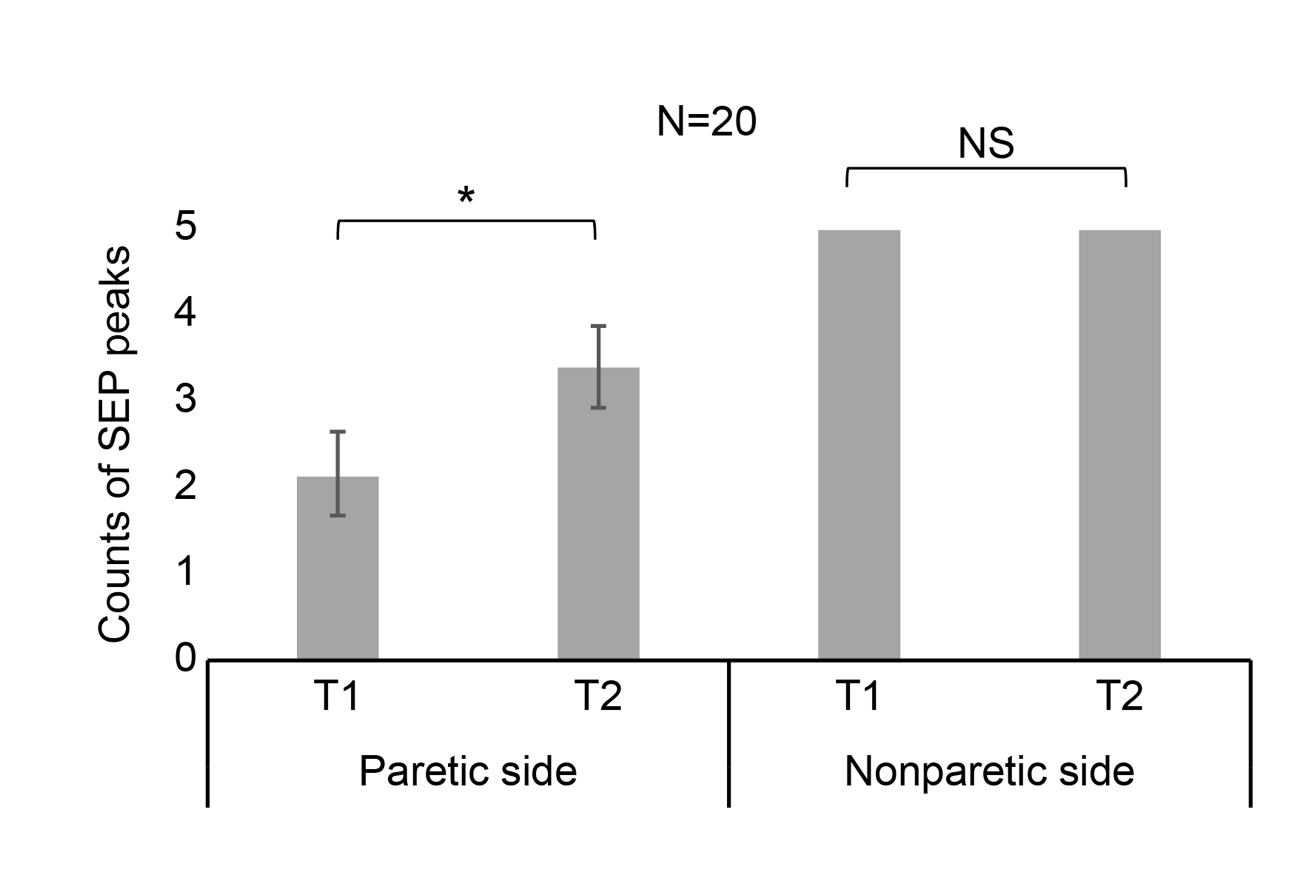


Figure S3: Features of SEPs in patients who underwent clinical assessment (Group B)

Graph showing the counts of median nerve SEP peaks after NI at T1 and T2 in patients who underwent clinical assessment (N=20). A significant increase is observed in the counts of SEP peaks at T2 (*: T1, 2.15 (0.49); T2, 3.4 (0.47); P*=*0.004; Wilcoxon signed-rank tests). All SEP peaks are preserved on the nonparetic side. All data are presented as mean (standard error of the mean).

NI: N20, SEP: somatosensory evoked potential, T1: initial assessment, T2: second assessment


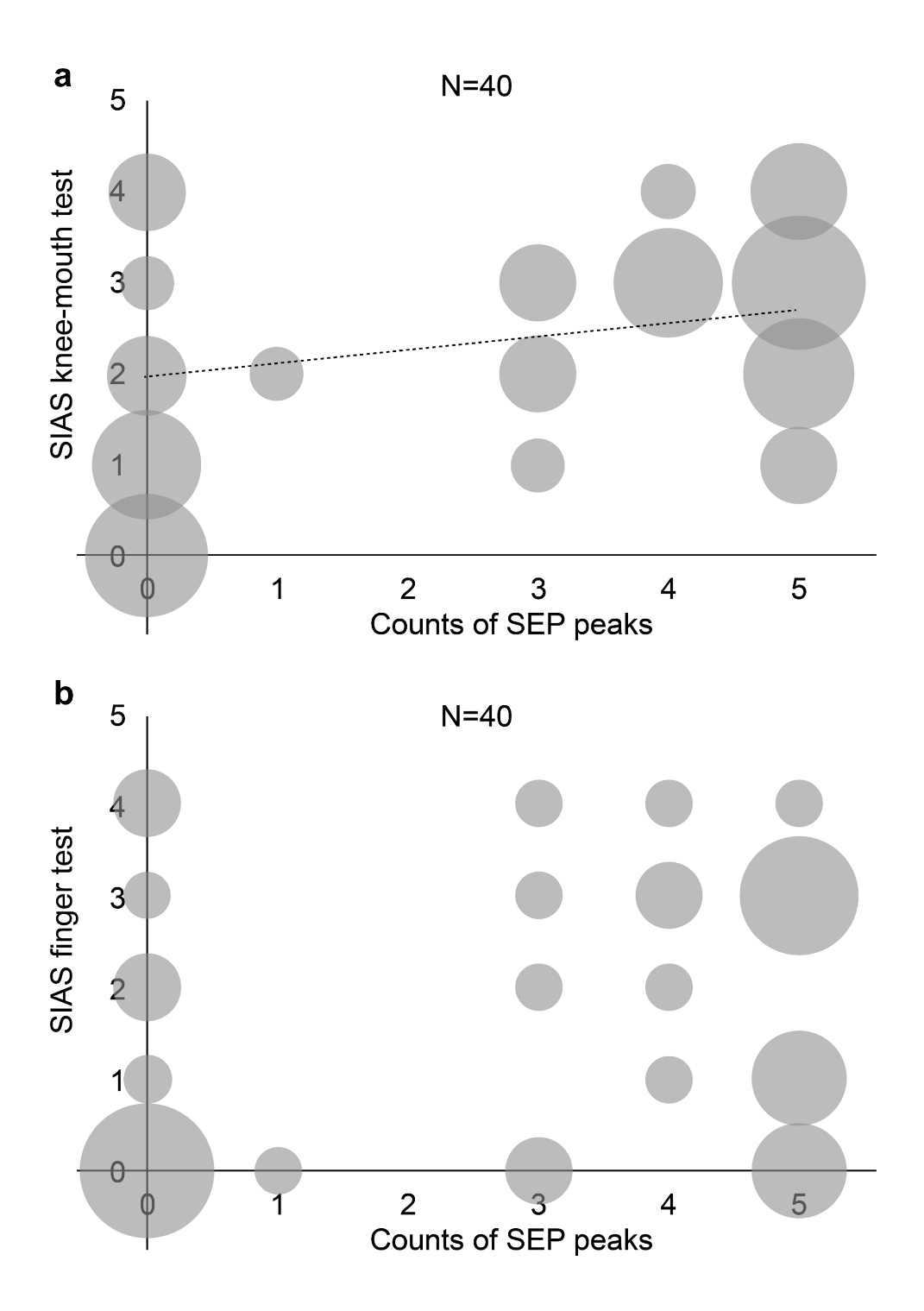


Figure S4: Scatter plot showing the correlation between SEP peak counts and each SIAS motor assessment (Group B)

(a) A moderate correlation is observed between SEP peaks and proximal upper limb motor function (ρ=0.44, P=0.004; Spearman’s rank correlation coefficient). (b) No correlation is observed between SEP peaks and distal upper limb motor function (ρ=0.22, P=0.178; Spearman’s rank correlation coefficient).

SEP: somatosensory evoked potential, SIAS: Stroke Impairment Assessment Set


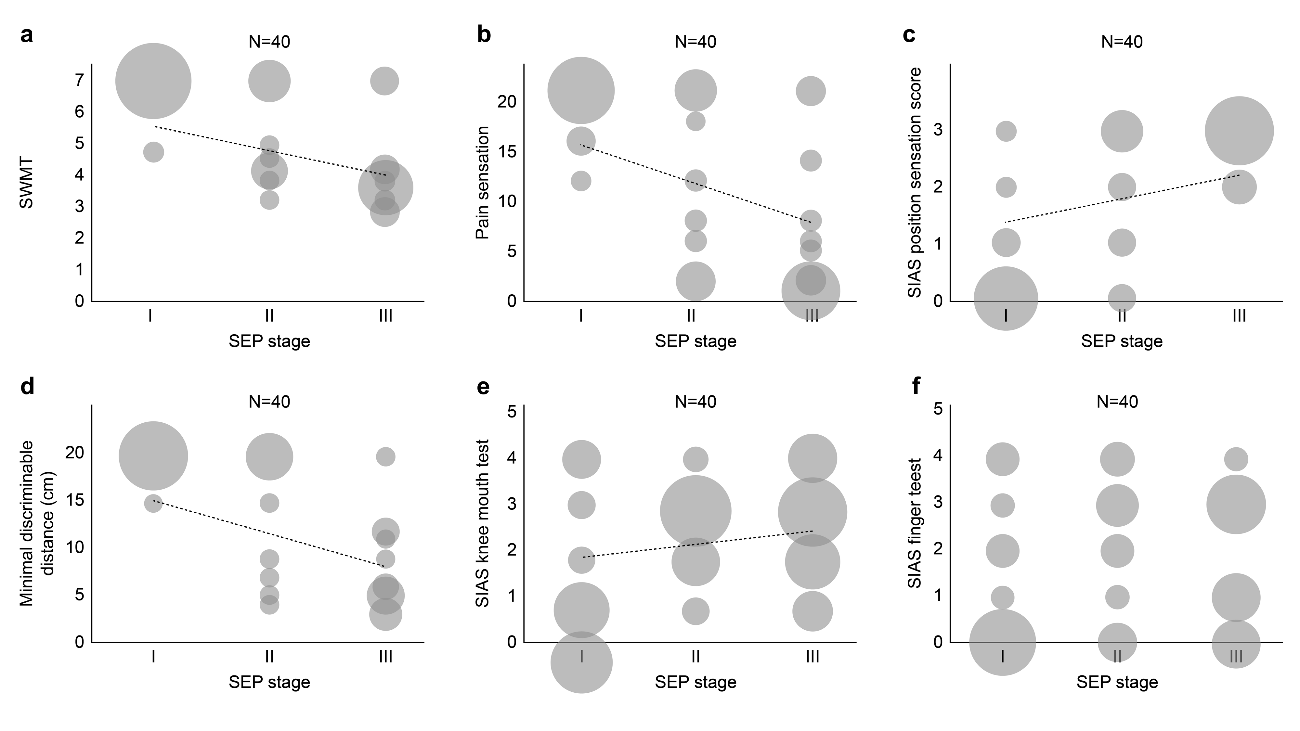


Figure S5: Scatter plots showing the correlation between the SEP stage and sensorimotor assessment (Group B)

Strong correlations are observed between (a) SEP stage and SWMT (ρ=-0.74, P<0.001; Spearman’s rank correlation coefficient), (b) pain sensation (ρ=-0.69, P<0.001; Spearman’s rank correlation coefficient), (c) SIAS position sensation (ρ=0.74, P<0.001; Spearman’s rank correlation coefficient), and (d) two-point discrimination (ρ=-0.75, P<0.001; Spearman’s rank correlation coefficient). (e) A moderate correlation is found between the SEP stage and proximal upper limb motor function (ρ=0.41, P=0.008; Spearman’s rank correlation coefficient). (f) No correlation is observed between the SEP stage and distal upper limb motor function (ρ=0.18, P=0.25; Spearman’s rank correlation coefficient).

SEP: somatosensory evoked potential, SWMT: Semmes–Weinstein Monofilament Test, SIAS: Stroke Impairment Assessment Set.

Table S1: Functional changes observed on the nonparetic side (Group B)

| Sensorimotor assessments | T1 | T2 | P-value |
| --- | --- | --- | --- |
| SWMT number | 3.56 (0.03) | 3.45 (0.07) | 0.120 |
| Pain sensation | 4.72 (1.17) | 3.95 (1.25) | 0.312 |
| Position sensation (SIAS) | 2.95 (0.05) | 2.95 (0.05) | 1.000 |
| Two-point discrimination | 4.65 (0.2) | 3.85 (0.22) | 0.008 |

All data are presented as mean (standard error of the mean).

T1: initial assessment, T2: second assessment, SWMT: Semmes−Weinstein Monofilament Test, SIAS: Stroke Impairment Assessment Set.

Table S2: Latencies of cortical peaks at T1 and T2 on the paretic side

|  | NI | PI | NII | PII | NIII |
| --- | --- | --- | --- | --- | --- |
| T1 | 20.24 (1.48) | 26.1 (2.96) | 32.91 (4.05) | 53.7 (5.83) | 90.04 (11.89) |
| T2 | 19.91 (1.47) | 26.52 (2.54) | 34.14 (3.56) | 53.12 (9.87) | 87.31 (15.12) |
| P | 0.023* | 0.25 | 0.90 | 0.029* | 0.61 |

All data are presented as mean (standard error of the mean).

NI: N20, PI: P24, NII: N33, PII: P45, NIII; N60, T1: initial assessment, T2: second assessment

Table S3: Interpeak latencies at T1 and T2 on the paretic side

|  | CCT | PI-NI | NII-NI | PII-NI | NIII-NI |
| --- | --- | --- | --- | --- | --- |
| T1 | 7.04 (1.48) | 6.22 (2.13) | 13.03 (3.56) | 33.43 (5.85) | 69.94 (12.51) |
| T2 | 7.05 (1.39) | 6.68 (1.88) | 14.31 (3.06) | 33.21 (9.63) | 67.56 (14.88) |
| P | 0.39 | 0.45 | 0.53 | 0.10 | 0.82 |

All data are presented as mean (standard error of the mean).

NI: N20, PI: P24, NII: N33, PII: P45, NIII; N60, CCT: central conduction time; T1: initial assessment, T2: second assessment

Table S4: Latencies of cortical peaks at T1 and T2 on the nonparetic side

|  | NI | PI | NII | PII | NIII |
| --- | --- | --- | --- | --- | --- |
| T1 | 19.23 (1.15) | 24.47 (1.86) | 30.34 (2.39) | 45.36 (4.53) | 78.95 (12.79) |
| T2 | 19.04 (1.09) | 24.4 (1.92) | 30.58 (2.26) | 45.43 (4.08) | 76.93 (12.3) |
| P | 0.021* | 0.66 | 0.41 | 0.90 | 0.22 |

All data are presented as mean (standard error of the mean).

NI: N20, PI: P24, NII: N33, PII: P45, NIII; N60, T1: initial assessment, T2: second assessment

Table S5: Interpeak latencies at T1 and T2 on the nonparetic side

|  | CCT | PI-NI | NII-NI | PII-NI | NIII-NI |
| --- | --- | --- | --- | --- | --- |
| T1 | 6.2 (0.74) | 5.23 (1.36) | 11.11 (2.08) | 26.12 (4.21) | 59.72 (12.64) |
| T2 | 6.11 (0.77) | 5.36 (1.4) | 11.55 (1.94) | 26.39 (4) | 57.89 (12.2) |
| P | 0.30 | 0.14 | 0.34 | 0.63 | 0.27 |

All data are presented as mean (standard error of the mean).

NI: N20, PI: P24, NII: N33, PII: P45, NIII; N60, CCT: central conduction time; T1: initial assessment, T2: second assessment

Table S6: Transition of a number of patients in each count of peaks from T1 to T2 on the paretic side

| Counts of peaks | 0 | 1 | 2 | 3 | 4 | 5 |
| --- | --- | --- | --- | --- | --- | --- |
| T1 | 22 | 1 | 2 | 4 | 5 | 13 |
| T2 | 15 | 0 | 1 | 2 | 6 | 23 |

T1: initial assessment, T2: second assessment.

Table S7: Transition of a number of patients in each SEP stage from T1 to T2 on the paretic side

| SEP stage | Ⅰ | Ⅱ | Ⅲ |
| --- | --- | --- | --- |
| T1 | 22 | 12 | 13 |
| T2 | 15 | 9 | 23 |

T1: initial assessment, T2: second assessment.

Table S8: Correlation between somatosensory evoked potential stage and sensorimotor assessments (Group B)

| Sensorimotor assessments | Correlation coefficient | P-value |
| --- | --- | --- |
| SWMT number | RS=-0.74 | <0.001 |
| Pain sensation | RS=-0. 69 | <0.001 |
| Position sensation (SIAS) | RS=0.74 | <0.001 |
| Two-point discrimination | RS=-0.75 | <0.001 |
| SIAS knee-mouth test | RS=0.41 | 0.008 |
| SIAS finger test | RS=0.18 | 0.25 |

SWMT, Semmes–Weinstein Monofilament Test; SIAS, Stroke Impairment Assessment Set
